# 3D Printed Temporary Veneer Restoring Autotransplanted Teeth in Children: Design and Concept Validation Ex Vivo

**DOI:** 10.3390/ijerph16030496

**Published:** 2019-02-11

**Authors:** Ali Al-Rimawi, Mostafa EzEldeen, Danilo Schneider, Constantinus Politis, Reinhilde Jacobs

**Affiliations:** 1 OMFS IMPATH Research Group, Faculty of Medicine, Department of Imaging and Pathology, KU Leuven and Oral and Maxillofacial Surgery, University Hospitals Leuven, 3000 Leuven, Belgium; dr.alirimawi@gmail.com (A.A.-R.); drdaniloschneider@gmail.com (D.S.); constantinus.politis@uzleuven.be (C.P.); reinhilde.jacobs@uzleuven.be (R.J.); 2 Department of Dentistry, Royal Medical Services, Jordanian Armed Forces, 00962 Amman, Jordan; 3 Department of Oral Health Sciences, KU Leuven and Paediatric Dentistry and Special Dental Care, University Hospitals Leuven,3000 Leuven, Belgium; 4 Department of Dental Medicine, Karolinska Institute, SE-171 77 Stockholm, Sweden

**Keywords:** CBCT, CAD/CAM, 3D printing, DLP, tooth autotransplantation

## Abstract

(1) Background: Three-dimensional printing is progressing rapidly and is applied in many fields of dentistry. Tooth autotransplantation offers a viable biological approach to tooth replacement in children and adolescents. Restoring or reshaping the transplanted tooth to the anterior maxilla should be done as soon as possible for psychological and aesthetic reasons. However, to avoid interfering with the natural healing process, reshaping of transplanted teeth is usually delayed three to four months after transplantation. This delay creates a need for simple indirect temporary aesthetic restoration for autotransplanted teeth. The aim of this study was to develop and validate a digital solution for temporary restoration of autotransplanted teeth using 3D printing. (2) Methods: Four dry human skulls and four dry human mandibles were scanned using cone beam computed tomography to create 3D models for 15 premolars. Digital impression of the maxillary arch of one of the skulls was captured by intra oral scanner. The digital work flow for the design and fabrication of temporary veneers is presented. The seating and adaptation of the 3D printed veneers were evaluated using stereomicroscopy and micro-computed tomography. (3) Results: Evaluation of the veneer seating using stereomicroscopy showed that the mean marginal gap at all of the sides was below the cut-off value of 200 µm. The overall mean marginal gap was 99.9 ± 50.7 µm (median: 87.8 (IQR 64.2–133 µm)). The internal adaptation evaluation using micro-computed tomography showed an average median gap thickness of 152.5 ± 47.7 (IQR 129–149.3 µm). (4) Conclusions: The present concept of using temporary veneers that are designed and fabricated with CAD/CAM (computer-aided design/computer-aided manufacturing) technology using a DLP (digital light processing) printer may present a viable treatment option for restoration of autotransplanted teeth.

## 1. Introduction

Computer-aided design/computer-aided manufacturing (CAD/CAM) use in dentistry started in the early nineties with subtractive manufacturing [1,2]. Recently, additive manufacturing technology, three dimensional (3D) printing, has been progressing rapidly and is applied in many fields of dentistry [3] such as: fabrication of surgical guides for placement of dental implants [4,5,6,7], guides for endodontic access and apical surgeries [8,9], fabrication of surgical guides and tooth replicas for tooth autotransplantation (TAT) [10,11,12], construction of physical models for orthodontics [13], manufacturing of dental implants [14], and study models, splints, and guides in orthognathic surgeries [15,16,17].

The process of 3D printing in dentistry goes through three stages, starting with data acquisition using low dose cone beam computed tomography (CBCT) [18,19] and/or intraoral scanner (IOS) [20,21], followed by processing and designing using dedicated software tools, and, finally, printing [12,22,23,24].

Different 3D printing technologies are being used in dentistry, which can be technically classified into: fused deposition modelling (FDM), stereolithography (SLA), PolyJet printing, MultiJet printing, ColorJet printing, selective laser sintering, and digital light processing (DLP) [3,24].

As aforementioned, CAD/CAM and 3D printing technology are already being applied in tooth autotransplantation (TAT) procedures [10,12]. TAT offers a viable biological approach to tooth replacement in children and adolescents after traumatic dental injuries (TDIs), agenesis, developmental anomalies, or specific orthodontic problems [25,26,27,28,29]. The treatment options available, for example implant placement, are limited by the ongoing dentoalveolar development [30], while orthodontic tooth alignment is challenging unless skeletal anchorage is applied [31,32]. TAT allows for periodontal healing and enables preservation of the alveolar ridge, maintaining the possibility of function and growth [25,33,34,35]. Restoring or reshaping the transplanted tooth to the anterior maxilla should be done as soon as possible for psychological and aesthetic reasons [36]. However, to avoid interfering with the natural healing process [33,37], reshaping of transplanted teeth is usually delayed three to four months after transplantation [38]. This delay creates a need for a simple indirect temporary aesthetic restoration for TAT, which so far has not yet been reported.

The aim of this study was to develop and validate a digital solution for temporary restoration of autotransplanted teeth using 3D printing technology.

## 2. Materials and Methods

### 2.1. Image Acquisition

Four dry human skulls and four dry human mandibles were scanned using a CBCT machine NewTom VGI EVO (QR Verona, Verona, Italy) Ethical Review Board of the University Hospitals Leuven (S55619 ML9535, University Hospitals Leuven). Scanning parameters were set for a standard mode, 360° rotation, 200 µm voxel size, and a field of view of 80 mm × 80 mm at 110 kV (x-ray tube voltage) and automatic tube current modulation. All data sets were exported using the Digital Imaging and Communications in Medicine (DICOM) file format with an isotropic voxel size of 200 µm and a slice interval and thickness of 200 µm.

### 2.2. Segmentation Protocol

CBCT scans were imported into MeVisLab (MeVis Medical Solutions AG, Bremen, Germany). Regions of interest including the single rooted first or second premolar were selected. All regions of interest images were normalized using an intensity windowing filter and then a median filter to suppress any noise and decrease confounding variables between the images.

**Figure 1:**
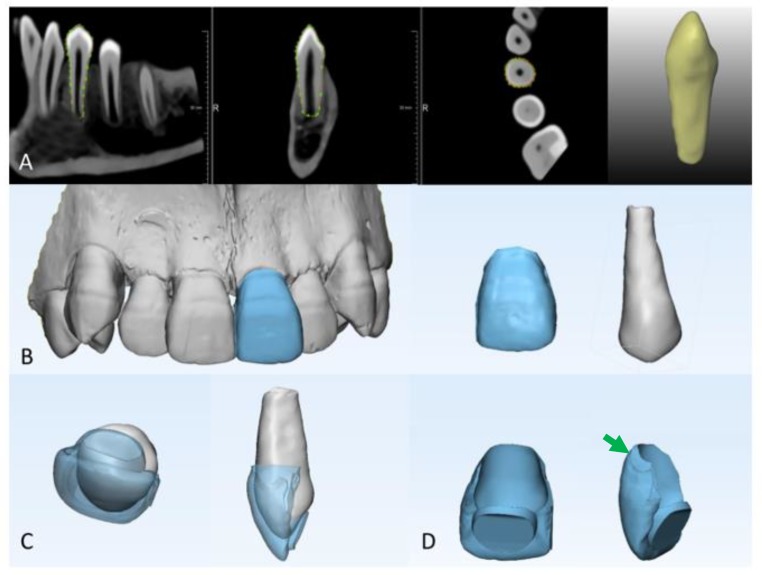
Digital flow for temporary veneer preparation. (**A**) Premolar segmentation; (**B**) designing temporary veneer based on the shape of the contralateral incisor (to mimic the clinical situation where one maxillary central incisor will be lost, a mirror image of the contralateral maxillary incisor was used to design the temporary veneer that will fit the transplanted premolar); (**C**) checking veneer thickness to ensure optimal printing; (**D**) final veneer design: removing undercuts, beveling the edges (green arrow), and inspecting the surface thickness is done to avoid print failure spots.

All single rooted premolars were then segmented using a dedicated tool that was developed in MeVisLab and validated for accurate tooth/root canal space segmentation as described [39] (Figure 1A). The tool applies interactive livewire boundary extraction to create a set of orthogonal contours around the tooth of interest. Livewire allows for a semi-interactive segmentation of structures with prominent edge image features [40]. Internally, the module generates a graph representation of the image to work on; the graph’s nodes represent image pixels and edges connect neighbouring pixels. The edges are weighted based on the cost function (image gradient magnitude). If starting and ending points are defined on such a graph, the shortest path (minimal cost path) is computed using dynamic programming (F * algorithm) [41]. This was followed by a variational interpolation algorithm that reconstructs the surface of an object with energy-minimizing, smooth, and implicit functions in order to create a 3D mask of the tooth surface (Figure 1A) [42].

After segmentation, the 3D triangle-based surfaces of the 15 premolars (four skulls: seven premolars, four mandibles: eight premolars) were reconstructed and saved as Standard Triangle Language (STL) files.

The digital impression of the maxillary arch of one of the skulls was captured by Trios IOS (Trios^®^ 3 Cart wired, 3Shape, Copenhagen, Denmark).

### 2.3. Designing of Temporary Veneers

The steps of veneer designing are illustrated in Figure 1. The digital 3D model of the maxillary central incisors was acquired using the Trios intra-oral scanner and the 3D models of the segmented premolars were imported to 3-matic version 12.0 (Materialise; Leuven, Belgium) (Figure 1B,C).

To mimic the clinical situation where one maxillary central incisor will be lost, a mirror image of the contralateral maxillary incisor was used to design the temporary veneer that will fit the transplanted premolar (Figure 1B). The crown of the central incisor was isolated from the 3D model which was created by the intra-oral scanner (Figure 1C) and was then moved to overlap the crown of the premolar. The transparency of the central incisor was then changed into medium transparency in order to control the thickness of the desired veneer during the design process (Figure 1C).

Subsequently, the 3D model of the premolar was subtracted from the 3D model of the maxillary central incisor (Figure 1D). The generated subtraction object represented the temporary veneer design; the design was further optimized and was then exported as an STL file, ready for printing (Figure 1D).

This process was repeated for the 15 3D models of the segmented premolars using the same maxillary central incisor. As a result, 15 digital models of temporary veneers were generated.

### 2.4. Three-Dimensional Printing of Temporary Veneers

The 3D models of the veneers and assigned premolars were exported to the Raydent studio software and were printed in a Raydent (RAM500, RayMedical, Seoul, South-Korea) DLP 3D printer. The printer utilizes liquid crystal planar solidification technology and was loaded with its specific resin material (crown and bridge resin).

**Figure 2:**
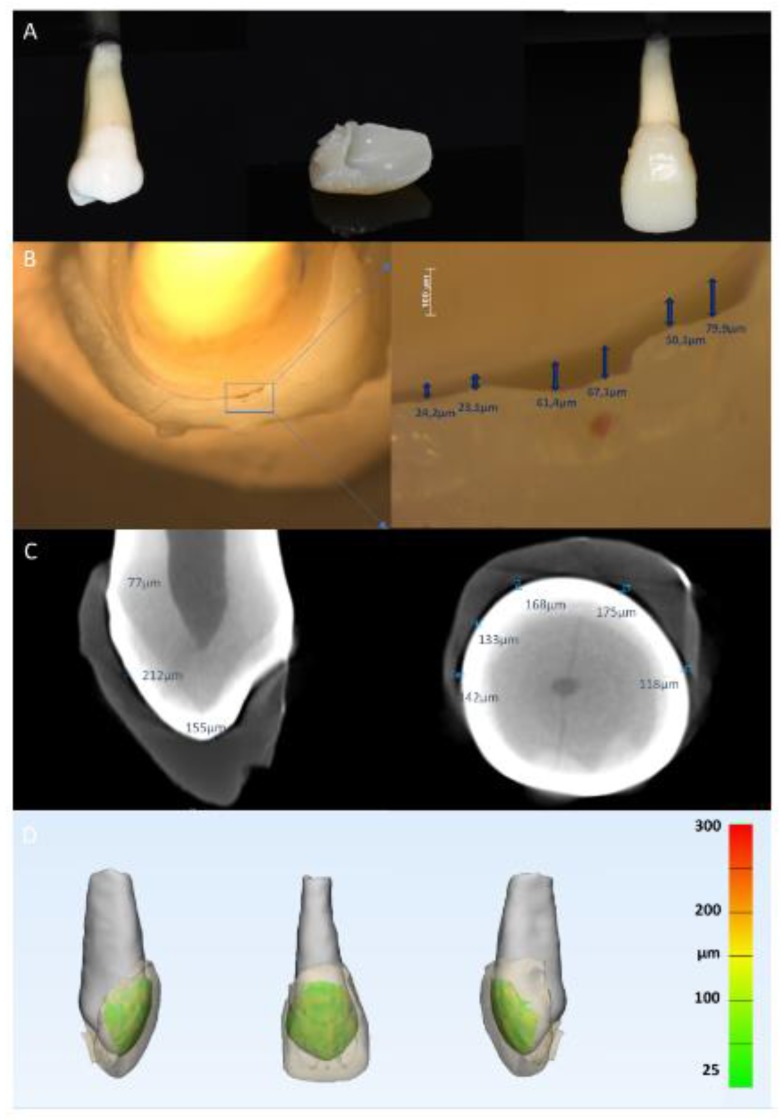
3D printed veneer and evaluation methods. (**A**) 3D printed veneer and fitting to premolar; (**B**) evaluation of veneer seating using stereomicroscopy; (**C**) internal gap evaluation using micro-computed tomography, D: quantitative analysis of gap thickness.

The printed veneers were cleaned in an ultrasonic bath using IPA 90% (Isopropyl alcohol) to remove residual resin and were then post cured with Curing Unit (RPC500). Figure 2A presents an example for the 3D printed temporary veneer.

### 2.5. Evaluation of Veneer Seating and Marginal Adaptation

All premolars were removed from the skulls and mandibles. Then, each veneer was seated onto its corresponding tooth. To ensure the correct seating of each veneer, corresponding to the designed position, a special holder was designed using in 3-matic version 12.0 (Materialise; Leuven, Belgium) and was 3D printed using the Connex printer (Object 360, Stratasys, MN, USA).

The cervical, mesial, and distal veneer margins were examined under a stereomicroscope (Olympus, Singapore) (Figure 2B). For each side, digital images for detected gaps were captured at 50 x magnification. Six measurements were made at each image resulting in 18 readings for each veneer.

### 2.6. Evaluation of Internal Adaptation Using Micro-Computed Tomographic Imaging

#### Image Acquisition

To check internal adaptation, the fitted veneers were scanned with the SkyScan 1172 micro-computed tomographic (µCT) system (Bruker, Antwerp, Belgium) (Figure 2C). The µCT parameters were 12.8 μm voxel size, 40 kVp, 250 mA, 0.5 mm aluminum filter, angular rotation step of 0.7°, 360° scanning, and exposure time of 0.295 s with a total scan duration of 22.5 min.

The x-ray projections were reconstructed using volumetric reconstruction software (SkyScan, Nrecon), beam hardening correction of 2%, and ring artefact correction were used for the reconstruction. Reconstructed slices were exported as DICOM files.

### 2.7. Segmentation Protocol, 3D Reconstruction, and Quantitative Analysis of Gap Thickness

To assess the internal adaptation, the gap between the veneer and the tooth was segmented using an indirect protocol applying logical operations. DICOM files of reconstructed images were imported into a dedicated tool which was developed in MeVisLab (MeVis Medical Solutions AG, Bremen, Germany).

The gap and enamel were segmented together as a single entity and were then saved as a binary image and as an intensity image. The intensity image was loaded, then the enamel was thresholded and saved as a separate binary image. The segmented binary images of the gap and enamel and the enamel separately were used to reconstruct two 3D models. The enamel 3D model was then subtracted from the 3D model of the gap and enamel, resulting in the 3D model of the gap (3matic, Materialise; Leuven, Belgium). The thickness of the resulting gap 3D model was then analyzed and expressed as a color-coded map (Figure 2D).

## 3. Statistical Analysis

Statistical analysis and graph plotting were performed using the statistical software package GraphPad Prism 7.00 (GraphPad Software, La Jolla, CA, USA). One-way way ANOVA was used to test for statistical differences at *p* < 0.05.

## 4. Results

**Figure 3:**
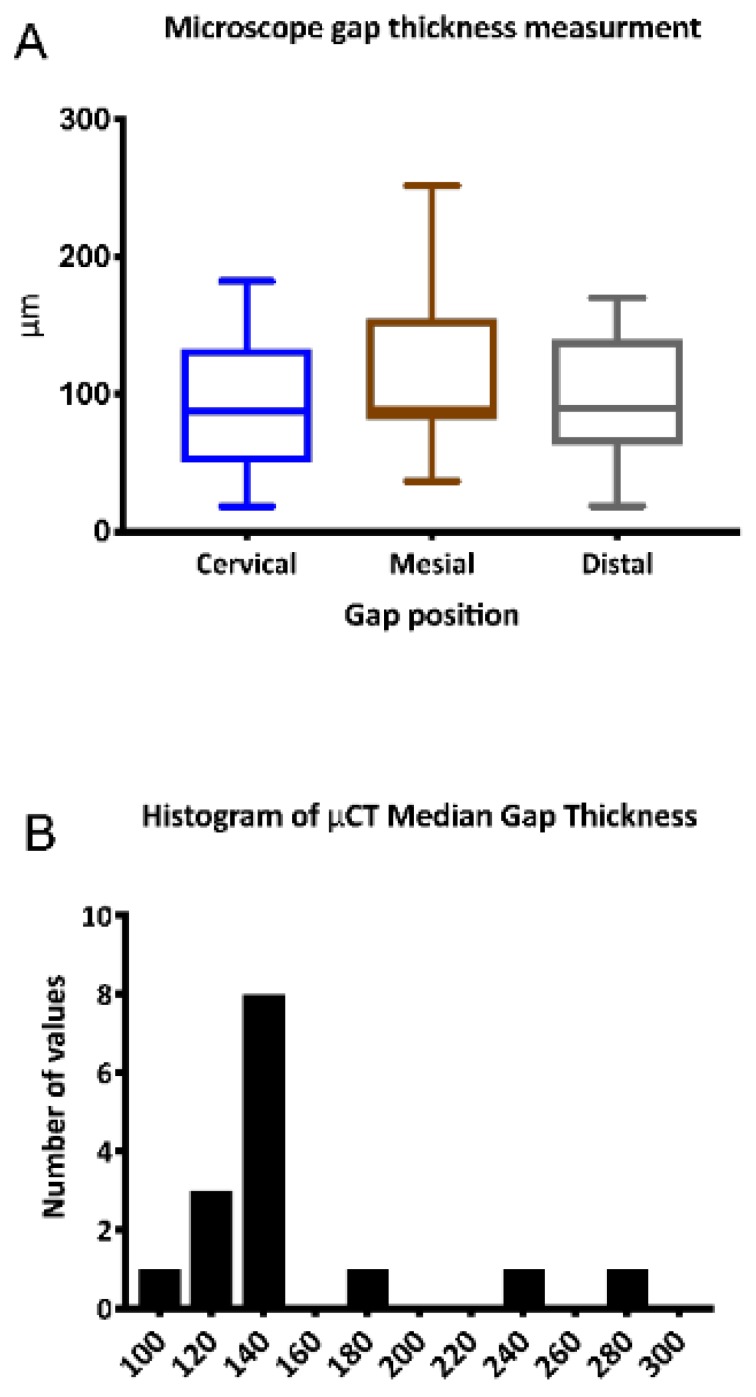
(**A**) stereomicroscopy gap measurements; (**B**) distribution of the gap thickness between the veneer and the tooth.

The resulting 3D printed temporary veneer is presented in Figure 2A. Evaluation of veneer seating using stereomicroscopy showed that the mean marginal gap at all sides was below the cut-off value of 200 µm (Figure 3A). The overall mean marginal gap was 99.9 ± 50.7 µm [median: 87.8 (IQR 64.2–133 µm)]. Cervically, the mean marginal gap was 92.4 ± 48.1 µm [median: 87.8 (IQR 50.2–133 µm)]. On the mesial side, the mean marginal gap was 111 ± 59.2 µm [median: 89.1 (IQR 81.8–155 µm)] and on the distal side, the mean marginal gap was 96 ± 44.9 µm [median: 89.8 (IQR 63.4–140 µm) (Figure 3A). Differences between the gap thickness measured at different positions did not show any statistically significant differences (*p* > 0.05).

Internal adaptation evaluation using μCT showed an average median gap thickness of 152.5 ± 47.7 (IQR 129–149.3 µm). Figure 2D presents a color-coded map for 3D gap thickness analysis demonstrating the homogenous thickness below the cut-off value of 200 µm. Moreover, the overall distribution of the gap thickness between the veneer and the tooth was below the cut-off value of 200 µm in the majority of the samples.

## 5. Discussion

Tooth autotransplantation TAT offers a viable biological approach to tooth replacement in children and adolescents after traumatic dental injuries (TDIs), agenesis, developmental anomalies, or specific orthodontic problems [25,26,27,28,29]. In a recent systematic review, Akhlef et al. [28] reported an overall survival rate for conventional TAT ranging between 93% and 100% (weighted mean: 96.7%, median: 100%) after 9 months to 22 years of observation (median: 8.75 years). The survival rates for conventional TAT of teeth with incomplete root formation was reported to be 97.4, 97.8, and 96.3% after 1, 5, and 10 years, respectively [29]. Studies reporting on the aesthetic results after TAT are limited [28]. Czochrowska et al. [38] reported a clinical assessment of a reshaped autotransplanted tooth using composite build-ups compared to a natural contralateral tooth according to objective parameters. The authors reported a 59% match, 27% deviation, and 14% mismatch with the natural contralateral tooth [38].

While restoring or reshaping transplanted teeth to the anterior maxilla is essential for psychological and aesthetic reasons [36], reshaping of transplanted teeth is usually delayed three to four months after transplantation to avoid interfering with the natural healing process [28,29,38,43]. This interval could be bridged using a simple indirect temporary aesthetic restoration for autotransplanted teeth.

The current report proposes a digital technique for designing and fabricating temporary veneers for autotransplanted teeth via chair side 3D printing technology, namely DLP. Simultaneously, the accuracy of this printing technology was validated by examining the marginal and internal adaptation of these temporary veneers.

A precise fit is an essential requirement for any dental restoration or prostheses. Ill-fitting prostheses will result in damage for the periodontium, tooth structure, and the prosthesis itself [23,44,45]. This study applied two methods for evaluating the adaptation of the veneers: direct view with microscope to measure the marginal fit, and µCT to evaluate the internal adaptation. Although there is no consensus about the best method to examine the marginal adaptation of fixed dental prosthesis, a direct view method is the most used method and with the most reproducible results. In the present study, marginal gaps were measured by stereomicroscopy (direct viewing method), resulting in a mean of a marginal gap of 100 µm, which is below the clinically acceptable value of marginal gaps of 120 µm [46,47].

To assess internal adaptation, µCT scans of the maxillary central incisor shaped veneers fitted on premolars, simulating the clinical situation, were used. The majority of the studies where µCT was used to assess internal adaptation performed two dimensional measurements of the internal gaps on cross sectional slides [48,49], while in this study, three-dimensional evaluation of the internal gap was applied. The average median thickness of the internal gaps in the present study was 152 µm, which is within the accepted values [50,51,52].

In a 2-year follow-up study on the use of 3D printed veneers, values of internal adaptation of porcelain laminate veneers ranged from 195 to 202 µm, which is higher than our values, while clinical performance was rated 100% satisfactory over the 2-year period [52].

One of the potential limitations of the suggested protocol is the use of one specific 3D printing technology and one specific printing material. Future studies are needed to study different 3D printing technologies other than DLP, while using other printing materials.

With the development of 3D printing technology and new resin materials developed specifically for dental restorations, it is expected that the use of this technique could be expanded for applications other than in autotransplanted teeth, especially in clinical situations where treatment should be done in a short amount of time, for example dental treatment under general anesthesia, trauma, and uncooperative children.

## 6. Conclusions

The present concept of using temporary veneers designed and fabricated with CAD/CAM technology using a DLP printer may present a viable treatment option for restoration of autotransplanted teeth. For the current design, values of marginal and internal adaptation were found to be within the clinically acceptable ranges.
